# Diurnal Curve of the Ocular Perfusion Pressure

**DOI:** 10.5005/jp-journals-10008-1195

**Published:** 2016-05-12

**Authors:** Fabio N Kanadani, TCA Moreira, BSP Bezerra, MP Vianello, J Corradi, SK Dorairaj, TS Prata

**Affiliations:** Chief, Department of Glaucoma, Medical Science University Hospital, Belo Horizonte, Brazil; Assistant Professor, Department of Retina, Medical Science University Hospital, Belo Horizonte, Brazil; Assistant Professor, Department of Glaucoma, Medical Science University Hospital, Belo Horizonte, Brazil; Assistant Professor, Department of Glaucoma, Medical Science University Hospital, Belo Horizonte, Brazil; Assistant Professor, Department of Glaucoma, Medical Science University Hospital, Belo Horizonte, Brazil; Assistant Professor, Department of Ophthalmology/Glaucoma, Mayo Clinic, Jacksonville, Florida, USA; Assistant Professor, Department of Glaucoma, Federal University of Sao Paulo Sao Paulo, Brazil

**Keywords:** Diurnal curve, Glaucoma, Ocular perfusion pressure.

## Abstract

**Purpose:** To describe the diurnal variation of the ocular perfusion pressure (OPP) in normal, suspects and glaucoma patients.

**Materials and methods:** Seventy-nine subjects were enrolled in a prospective study. The diurnal curve of intraocular pressure (IOP) was performed and blood pressure measurements were obtained. Each participant was grouped into one of the following based upon the clinical evaluation of the optic disk, IOP and standard achromatic perimetry (SAP): 18 eyes were classified as normal (normal SAP, normal optic disk evaluation and IOP < 21 mm Hg in two different measurements), 30 eyes as glaucoma suspect (GS) (normal SAP and mean deviation (MD), C/D ration > 0.5 or asymmetry > 0.2 and/or ocular hypertension), 31 eyes as early glaucoma (MD < -6 dB, glaucomatous optic neuropathy and SAP and MDs on SAP. Standard achromatic perimetry was performed with the Octopus 3.1.1 Dynamic 24-2 program. Intraocular pressure and blood pressure measurements were taken at 6 am, 9 am, 12, 3 and 6 pm. The patients stayed in the seated position for 5 minutes prior to blood pressure measurements.

**Results:** The mean IOP values in all groups did not follow any regular pattern. The peak IOP was found to be greater in suspect [18.70 ± 3.31 (mm Hg ± SD)] and glaucoma (18.77 ± 4.30 mm Hg) patients as compared to normal subjects (16.11 ± 2.27 mm Hg). In studying the diurnal variation of the OPP, we found lower values at 3 pm in normals (34.21 ± 2.07 mm Hg), at 9 am in suspects (54.35 ± 3.32 mm Hg) and at 12 pm in glaucoma patients (34.84 ± 1.44 mm Hg).

**Conclusion:** Each group has a specific OPP variation during the day with the most homogeneous group being the suspect one. It is important to keep studying the IOP and OPP variation for increased comprehension of the pathophysiology of glaucomatous optic neuropathy.

**How to cite this article:** Kanadani FN, Moreira TCA, Bezerra BSP, Vianello MP, Corradi J, Dorairaj SK, Prata TS. Diurnal Curve of the Ocular Perfusion Pressure. J Curr Glaucoma Pract 2016;10(1):4-6.

## INTRODUCTION

The two main theories for pathogenesis of the glaucomatous optic neuropathy (GON) are vascular and mechanical. Both have been defended by research groups on the last 150 years. In congenital, acute angle close and secondary glaucoma, clearly the increase in intraocular pressure (IOP) is enough to cause GON. However, innumerable findings of normal tension glaucoma (NTG) cannot satisfactorily be explained only by this theory. The fact is that the reduction of the ocular blood flow (OBF) frequently precedes the structural damage. It also occurs in other parts of the body of these glaucomatous patients, indicating that hemodynamic adjustments can at least partially be primary. The biggest cause of this reduction is not atherosclerosis, but a vascular disequilibrium that leads to the reduction of the ocular perfusion pressure (OPP) and insufficient autoregulation.^1^

The reduced OBF can have a decisive implication in the pathophysiology of many ocular illnesses like diabetic retinopathy, age-related retinopathy, pigmentary retinosis, myopia and glaucoma. Abnormalities of the blood flow in glaucoma have been evaluated with diverse techniques, including angiofluoresceinography, colored Doppler, Doppler laser fluxometry and pulsatile ocular blood flow (POBF) tonograph.^2^ Using the angiofluoresceinography Hayreh et al^3^ demonstrated a zone of the short posterior ciliary’s arterioles that supply the posterior portion of the optic nerve. This area can be vulnerable to the ischemia when the OPP is reduced. The OPP is defined as 2/3 of median arterial pressure (MAP) less IOP (OPP = 2/3 MAP-IOP).^4,5,11^

In agreement with the vascular theory, low systemic arterial pressure relative to IOP can lead to a low OPP, consequently, decreasing the perfusion of the optic nerve and leading to a glaucomatous loss of the visual field. On the contrary, systemic hypertension can increase the risk of damage to the small vessels of the optic disk.^5^ Leske MC (Leske Arch, 2004)^5^ studying the relationship between blood pressure and glaucoma reported increased prevalence of glaucoma when the diastolic perfusion pressure fell below 55 mm Hg. A reduction in MAP or an increase in IOP can diminish the ocular perfusion, but the accurate mechanism of the regulation of the IOP is unknown. If the autoregulation mechanisms are continuous, the sanguineous flow will remain steady with a substantial fall of the ocular perfusion.^4^

The aim of this study was to determine the OPP variation during the day in normal, suspects and glaucoma patients.

## MATERIALS AND METHODS

Prospective and comparative study in which 79 eyes were enrolled. All patients were recruited in the Santa Casa’s Eye Clinic of Belo Horizonte. The normal individuals were volunteered friend or parents of the suspects and glaucoma patients. Informed consent was obtained from all participants following the Tenets of the Helsinki’s declaration.

The diurnal curve of IOP was performed and blood pressure measurements were obtained. Each participant was grouped into one of the following based upon the clinical evaluation of the optic disk, IOP and standard achromatic perimetry (SAP): 18 eyes were classified as normal (normal SAP, normal optic disk evaluation and IOP < 21 mm Hg in two different measurements), 30 eyes as glaucoma suspect (GS) (normal SAP and mean deviation (MD), C/D ration > 0.5 or asymmetry > 0.2 and/or ocular hypertension), 31 eyes as early glaucoma (MD < -6 dB, glaucomatous optic neuropathy and SAP and MDs on SAP. Standard achromatic perimetry was performed with the Octopus 3.1.1 Dynamic 24-2 program. Intraocular pressure and blood pressure measurements were taken at 6 am, 9 am, 12, 3 and 6 pm. The patients stayed in the seated position for 5 minutes prior to blood pressure measurements.

The statistical analysis was performed with the Statistical Package for the Social Sciences (SPSS) 10.1 (SPSS Inc. Chicago, IL, EUA). Results were expressed as mean ± standard deviation and paired Student’s t-test was used to evaluate the level of significance. A p value of 0.05 or less was considered significant.

## RESULTS

**Graph 1:**
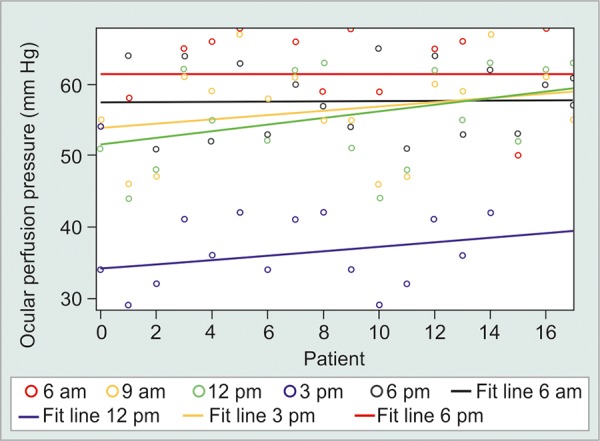
Diurnal curve of IOP and OPP in normal patients

**Graph 2:**
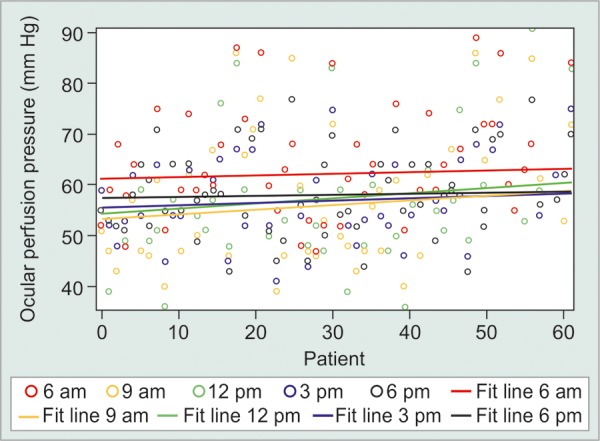
Diurnal curve of IOP and OPP in glaucoma suspect patients

**Graph 3:**
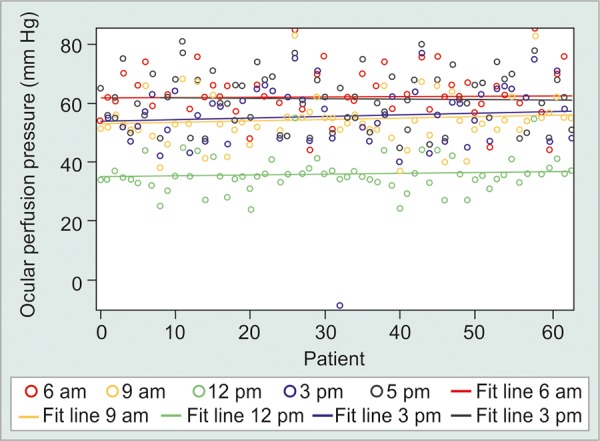
Diurnal curve of IOP and OPP in glaucoma patients

The mean IOP values in all groups did not follow any regular pattern. The peak IOP was found to be greater in suspect [18.70 ± 3.31 (mm Hg ± SD)] and glaucoma (18.77 ± 4.30 mm Hg) patients as compared to normal subjects (16.11 ± 2.27 mm Hg). In studying the diurnal variation of the OPP, we found lower values at 12 pm in normals (58.77 ± 10.45 mm Hg), (Graph 1) at 9 am in suspects (51.69 ± 12.69 mm Hg) (Graph 2) and at 9 pm in glaucoma patients (48.96 ± 8.91 mm Hg) (Graph 3).

All the OPP values were higher in the control group in all times in comparison to suspects and glaucoma patients. The same was found in suspects in comparison to glaucoma patients.

## DISCUSSION

Leske et al^5^ investigating the relationship between OPP and incidence of open angle glaucoma (OAG) found a relative risk of 3.1 for patients with OPP < 41.0 mm Hg. Fuchsjäger-Mayrl et al^11^ reported that the hemodynamics of ocular parameters are lower in patients with OAG in comparison with normal patients. The diurnal changes of the IOP were similar in normal and POAG patients; however, the OPP was significantly different between these groups with significant reduction after lunch and presenting lesser value at 7 am of the morning. Our results had demonstrated an OPP of 60.45 mm Hg (SD 10.9) in normal patients, of 53.18 (SD 11.56) in suspects and of 51.96 mm Hg (SD 9.59) in glaucomatous patients.

In patients with untreated glaucoma, the diurnal change in IOP has been reported to be between 4.8 and 18.4 mm Hg, whereas, in the normal eyes, it has been reported to be between 2.8 and 6.5 mm Hg.^6,7^ In our normal group, the IOP fluctuation was 1.67 mm Hg. The same stability was found in our suspects (0.50 mm Hg) and in our glaucoma group (1.71 mm Hg).

Under physiological conditions, low perfusion pressure is compensated by low resistivity to flow (autoregulation). There are indications that this behavior might be altered in some subjects. It has been demonstrated that otherwise healthy subjects with vasospastic syndrome, do have altered blood flow regulation in their central retinal artery, as measured by color Doppler imaging (CDI).^8^ This renders ocular perfusion directly dependent on perfusion pressure. Interestingly, a very similar behavior is observed in patients with progression despite normal or normalized IOP,^9^ indicating that damage may occur if low perfusion pressure is combined with abnormal or insufficient autoregulation. All these observations indicate that vascular regulation or dysregulation, respectively, might be key factors in the pathogenesis of GON.

There is evidence of an abnormal association between ocular perfusion parameters and systemic blood pressure in patients with glaucoma. However, these data refer to long-term adaptations of perfusion to the eye with blood pressure rather than a short-term increase in blood pressure.^10^ Despite this, we cannot quantify with any current diagnostic method, the real amount of blood in the anterior portion of the optic nerve, neither the level of existing gaseous exchange in this place. We also do not have a reliable technique to measure the blood flow in the anterior portion of the optic nerve. It is important to point out that although were not found any statistically significant difference on the OPP during the day, the absolute OPP value was lower in the glaucoma group in comparison to suspects and normals.
